# Exploring the Experiences of Sexual Violence/Abuse Survivors Attending a Recovery Group: A Qualitative Study of Recovery and Evaluation Implications

**DOI:** 10.1177/10778012231202999

**Published:** 2023-09-26

**Authors:** Jane Meyrick, Amelia Anning

**Affiliations:** 1 1981 University of the West of England, Bristol UK

**Keywords:** sexual violence, groups, qualitative, evaluation

## Abstract

Sexual violence/abuse (SV/A) is increasingly recognized requiring improved, evidence-based treatments. Delivery of group-based support in survivor services is common but often lacks evaluation. This qualitative study explored how survivors experienced groups and wanted recovery measured. Semistructured interviews with survivors of SV/A from two groups (*N* = 13, female: 25–64 years: mainly White British, heterosexual) were analyzed using an inductive approach to thematic analysis. Three overarching themes were identified including “anger,” recovery “in the company of” others and “different destinations.” The work contributes recognition of the need for evaluation with meaning to survivors and the potential for anger to be used in group activism.

## Introduction

One in five women and one in 20 men have experienced sexual assault since the age of 16 in the UK (Stripe, 2021a), and rates of sexual offences reported to the police have tripled in recent years (Stripe, 2021b) set against a picture of sustained global rates (Borumandnia et al., 2020) and an intersectional pattern of victimization (Armstrong et al., 2018).

Experiencing sexual violence can have devastating consequences both immediate and the long term. Issues experienced may be physical including sexually transmitted infections, gynecological/reproductive problems, sleep disorders, and gastrointestinal symptoms (Ades, 2020). Longer-term mental health outcomes among victims/survivors are also poor including depression, anxiety, and posttraumatic stress disorder (PTSD) compounded by coping mechanisms such as substance misuse, self-harm, and suicide (Brooker et al., 2018; Dworkin et al., 2017). The human cost is hard to capture, the UK Home Office projected the total socioeconomic cost of 122,000 rape offences in 2015/16 as £4.8 billion (Heeks et al., 2018) increasing to £12.5 billion with the addition of other sexual offences (based on the Crime Survey England and Wales).

Formal support services for victims/survivors vary and include a mixture of state funded or grassroot charity based services. Centrally funded sexual assault referral centers (SARCs), allied to the criminal justice process and a network of mixed-funded charitable rape crisis organizations, provide support in the UK whereas in the USA, systems have evolved state by state from grassroots organizations with variable state funding (Iowa Coalition, 2010). UK provision of specialist sexual violence support appears highly variable and the subject of a current UK mapping exercise (Combes et al., 2019). Rigorous empirical evaluation (powered, comparative design, control groups, longer-term impact, and inclusive of minority groups) of based sexual assault interventions is limited (Heard & Walsh, 2023) but improving (Brown et al., 2019). Older systematic reviews (Regehr et al., 2013; Vickerman & Margolin, 2009) evidence mixed quality evidence around the effectiveness of cognitive processing therapy, prolonged exposure therapy, stress inoculation therapy, and eye movement desensitization and reprocessing in reducing measures such as PTSD, depression, anxiety, feelings of guilt, and experiencing dissociation with some more recent mixed evidence for early intervention (Lomax & Meyrick, 2020).

Evidence specific to group interventions shows their increasing use as the delivery mechanism for either support or trauma-focused therapeutic interventions in a scoping review of studies on group interventions for adult survivors (Heard & Walsh, 2023), which found only 11 studies, using standardized measures including PTSD, depression symptoms, anxiety, guilt, dissociation, and drug and alcohol use plus some qualitative evaluation methods. They concluded that more rigorous evaluation research was needed including a better understanding of the process of recovery. One review (Schwartze et al., 2019) found evidence of the effectiveness of group delivery of Cognitive Behavioural Therapy (CBT), and a thematic review of eight studies (Konya et al., 2020) found evidence of effectiveness for peer-led groups for survivors of sexual abuse/assault was of low quality but suggestive of benefits through interpersonal mechanisms such as mutuality and connectedness or understanding. Elements of group approaches that address survivors’ stigma, shame, and silence through building trust and relationships need to be explored through research on how this is experienced, expanding on existing experience-related evidence of wider interventions (Brown et al., 2022).

Evaluation practice and the resulting evidence base put the spotlight on issues of power, who defines or measures success, and how this reflects what is valued. Collated survivors’ experiences of interventions go beyond mental health measures of effectiveness to include physical health, mood, understanding of trauma, and interpersonal/social/work rehabilitation (Brown et al., 2022). Trauma-informed approaches to sexual assault recovery highlight the need for interventions to focus on giving power back to survivors (DePrince & Gagnon, 2018), and how interventions measure and evaluate success also contributes to that process.

This study aimed to fill the gap in the evidence around survivor-informed approaches to the experience of change within groups and how surviviors experiencing groups felt success should be measured, judged, and evaluated. Carried out through exploratory qualitative interviews with women who had attended two specialist sexual abuse service groups, the work informs practice, how groups work and how this connects with current evaluation practice.

## Methods

This study used a qualitative methodology to obtain a deeper insight into individual experiences and perspectives of sexual violence recovery groups and evaluation of that experience.

This approach provided victims/survivors with a voice and central contribution to the discussion around how services for them could be evaluated. Participants were recruited from two “justice groups” located in different areas in the south-west of England run by Somerset and Avon Rape and Sexual Abuse Services (SARSAS). The groups ran over 10 sessions and provided a nurturing space to help understand sexual violence and its impact on different responses to trauma, boundaries and relationships, self-esteem and self-care, and moving forward. This was done with a mixture of group activities and discussions and included arts-based, self-care activities. COVID restrictions meant the later sessions were delivered online via TEAMS. All 18 group participants agreed to take part in the study, and 13 participants were ultimately interviewed between February and March 2021. Ethical approval for the study was secured from the University of the West of England (HAS.20.07.200) (Table 1).

**Table 1.** Participants.

Age	Sexual abuse experience	Sexual orientation	Ethnicity	Gender
63	Adult nonrecent	Heterosexual	White British	Female
57	CSA	Bisexual	White British	Female
46	CSA	Heterosexual	White British	Female
57	Adult nonrecent	Heterosexual	White British	Female
50	CSA and nonrecent adult	Not asked	White British	Female
28	CSA	Heterosexual	White British	Female
34	Adult nonrecent	Bisexual	Mixed Heritage	Female
62	CSA	Not asked	White British	Female
42	Adult nonrecent	Heterosexual	White British	Female
55	Adult nonrecent	Heterosexual	White British	Female
48	Adult nonrecent	Bisexual	White British	Female
28	Adult recent	Heterosexual	White British	Female
29	Adult nonrecent	Not asked	Not asked	Female

*Note*. CSA, child sexual abuse.

### Procedure

The interviews were conducted online (video call via Microsoft TEAMS), a format participants were familiar with. All participants were provided with full information about the study in an information sheet and consented in writing to the interviews/recordings, supportive referral information was also given. Interviews lasted generally around 1 h and were then transcribed and anonymized. Consent forms were returned by email prior to the beginning of the interview, and all participants were offered support follow-up with SARSAS who carried out the initial recruitment among their own client group. All 18 group participants volunteered for the study, but five could not make the interview times offered within the research project window. Prevention of secondary trauma within researchers (Williamson et al., 2020) was seen as a priority by the research team, and the primary interviewer was offered restorative supervision plus specialist counseling support on demand.

### Materials/Interview Schedule

The interview schedule was semistructured in order to facilitate a more open discussion of research questions and enabled other relevant issues to be raised by participants or pursued by the researcher. It covered research questions reflecting the aims of the study including open questions about participants’ experience of the group, the impact of participation, improvements that could be made, and both how participants conceptualized success and how this could be measured. All interviews were carried out by the research assistant (AA).

### Data Analysis and Theoretical Stance

Once transcribed, the interviews were analyzed using thematic analysis in order to identify key themes; primary data analysis and coding were carried out by the lead author and the research assistant following an inductive approach, moving from data or “from the ground up” to construct codes, which were then grouped and recast into themes (Glaser & Strauss, 2017). Measures to establish rigor (Meyrick, 2006) included an audit trail of quotes to theme (example table of all quotes/codes for theme 'anger' included in Table A1 see the appendix). The work is underpinned by an understanding of intersectional theory (Armstrong et al., 2018). The primary analyst (JM) is an experienced sexual violence researcher and as such brings knowledge of the literature and practice context to her construction of key themes within an individual reflexive reading of accounts (Braun & Clarke, 2019; Meyrick, 2006). Primary analysis was discussed with the second author and with the specialist support provider (SARSAS). No deviant case analysis (Meyrick, 2006) was necessary.

### Participants and Recruitment

The participants had generally experienced nonrecent sexual abuse/violence or child sexual abuse, were female, aged between 28 and 63 years, were mainly White, and whose sexual orientation where known was mainly heterosexual or bisexual. All were initially approached and recruited via SARSAS who provided with a participant information sheet and the opportunity to ask questions.

All group participants completing a 'justice group' program the year prior to the study at two locations were sent study information and an invitation to participate; all 18 members volunteered, and 13 were finally interviewed online as COVID restrictions prevented face-to-face meetings. Five were unable to find a mutually agreeable slot within the data collection window; as such, the sampling strategy of all participants in both groups was felt to adequately capture their experience with some consideration of systemic reasons for later dropout.

## Results

**Figure 1.**
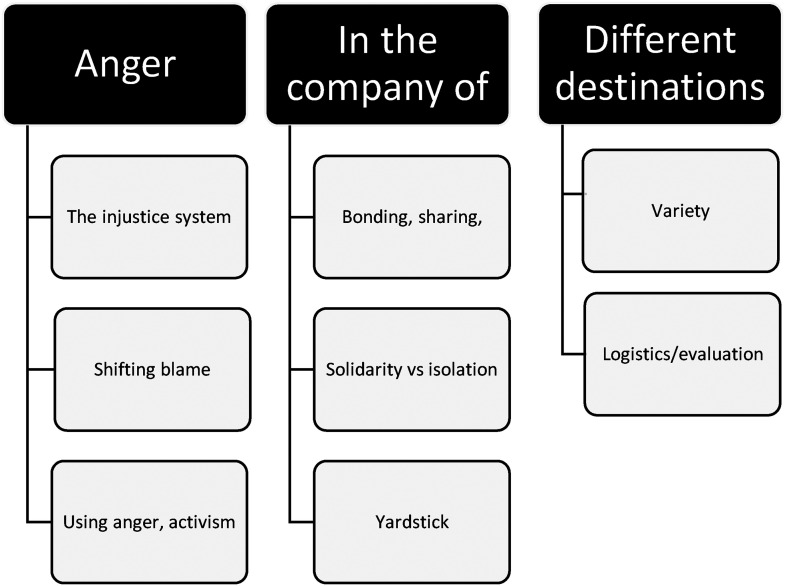
Overview of themes.

This section presents the higher-level conceptual thematic analysis. An overview of the three key themes is represented in the diagram, and each theme is broken down into supporting subthemes (Figure 1).

### Anger: “Because We Are All Angry About It” (OJG08)

A dominant and somewhat unexpected theme was characterized by participants’ anger, both about the initial trauma and, importantly, in relation to their subsequent treatment, often within the criminal justice system. Within the context of the research question, the focus of that anger was seen as a key indicator of recovery.

Most participants talked about anger in relation to experiencing injustice, losing their voice, or a lack of general accountability.

I’ve spent many years where I just felt horrible, sad, unhappy, guilty, ashamed but not angry when I would like to feel angry … I think that's a healthy way of recognising that that wasn’t right. (JG18)

The sense of injustice prevents us from carrying on with our lives. (OJG12)

That experience of injustice for some increased the burden of self-blame, but many felt that the group helped them *shift that blame* from themselves toward the perpetrators and the systems that had not held perpetrators accountable for the harm, hence the concept of the *injustice system*.

There was this idea that maybe it was my fault that I didn’t get justice … after listening to the other women I realised the problem is of a different nature, is not something I did wrong … I was able to put the responsibility where it belonged. (OJG12)

It's helped me come to terms with the idea that actually there is no justice… I think now the ability to, to see through it, to see past it, to see beyond. … It's not my failure. So it's like there is light after, there is life after the darkness and failure. (JG04)

So much so that some saw the *shifting of blame* and fault as a key purpose of the groups; “the main goal is, its not your fault, it is definitely not your fault”. (JG02)

In other words, recovery was seen as working toward the point of recognizing that it was someone else's fault, “I was able to put the responsibility where it belongs you know with the perpetrators the institutions that support them instead of supporting us” (OJG12).

The next step for many was using that anger to engage in activism or resistance. “Success would be, you know, if I was helping on some campaign, you know, doing something or success would be to help other women. Yeah… and success would be for me to go global. Attend a big summit. You know, be part of something” (OJG17).

This focus on anger and directing it into activism was something recovery groups can actively build but also speaks directly to sexual violence as a tool for stuctural gender inequality. Therefore, addressing systemic justice failures through activism is a role participants in this study wanted. It was recovering something that was lost through taking part in the fight against inequality and resisting the perpetration of sexual violence and subsequent legal impunity. Furthermore, they saw this shift away from an internal focus of blame as a measure of recovery.

### In the Company of Others

This theme picked out the contribution of the group itself to participants’ understanding of their own recovery. For many women attending, there was clear value placed on *bonding and sharing* with others and part of that came from acceptance and belief or validation of their experience from others. The feelings of sharing experience in turn opened up a pathway to more positive ways of coping including greater self-compassion.

I thought if I keep quiet it will all go away, I pushed it down inside … talking about things in/ through the group made we realise we’ve done the same thing. (JG07)

Forward rather than backwards. (JG02)

To discover that there's a sort of mixed feeling of amazement that you can be fitting in and understood. (OJG17)

The *solidarity* gained through telling and being heard was felt to redress the previous silence and isolation that many had experienced, it was regaining community, and it was combating *isolation*. “The most important thing is that I am treated with respect, validated, that my opinions and what I've experienced is valid and that I am believed” (JG18). “Slowly realises that you're not on your own and you're with other people and they've all got something to say” (OJG14).

The contribution of expert facilitation was recognized and continued the idea of doing something together, with others. “They [facilitators] didn’t try to fix us, they were there to be *in the company of us*” (OJG12).

That *solidarity* was expressed by some women as a normalizing of their own trauma responses: “the enormity of the fact that actually what happens in your brain is shared by everybody who undergoes that kind of experience… very reassuring” (OG17).

For many, an important part of doing something with a group was the way they could either see themselves or see their progress by measurment against others on the same journey, using them as a *yardstick*.

I would listen to people share and think, well, at least I haven’t got PTSD, thank goodness, how awful for them, and then for me, it was like a slow dawning realisation that I did, yeah. (OG17)

It helped me, it acted as a *yardstick* for me to think … well I'm not as badly off as some of those are and my experience isn't as bad as say, that Lady… it's worse than that one but not as bad as hers… but I don't actually feel stupid anymore. (OJ07)

Therefore, the role of these companions can be seen as a group to witness, believe, and normalize and, in so doing, enable the individuals to move forward in a way that a wider lack of voice, recognition, and belief had hampered. Part of that was the mirror others provided in seeing your own progress but also needs to be understood in the context of the wider silencing of sexual violence and abuse, the need to reconnect having been shut out.

### Different Destinations

Part of the aim of this research was to inform specialist services about how to best capture progress in the evaluation of groups as an intervention for survivors. Central to this was our goal of putting the survivors understanding of their own progress at the heart of evaluation practice and the need for the evaluation process to mirror the restorative processes of the group, giving voice, survivor led, etc.

Evaluation practice tends to focus on standardized delivery and standardized measures. The challenge is that within these groups, what participants saw as success *varied* and was highly individualized. Some felt what was important to them was the process of regaining their voice and leaving behind silence and, in doing so, regaining both confidence and power: “Being more open and honest and brave about speaking the truth about yourself and being okay being yourself” (OJG08). “I thought if I kept it quiet, it’d all go away” (JG07).

Others were more focused on reconnecting in order to make progress through addressing their feelings of isolation after experiencing sexual violence and abuse, “to get on with my life, to find a sense of community” (JG04).

Moving past previous feelings of shame and worthlessness were also understood as an indicator of progress.

I don’t feel ashamed of myself anymore, that's such a huge thing. I don’t feel like I’m dirty or I’m horrible. (OJG9)

You feel so degraded and worthless, so to actually feel like you are worthy as a person and as a human being is a real big achievement. (OJG19)

A very concrete outcome for some was something around regaining their sense of self-identity and losing the “victim” identity. “I wasn’t going around in public with like victim written on my forehead” (JG07).

How you might see these elements captured in evaluation measures of progress was expressed in a *variety* of ways including the following: “planning for the future” or “change” or “taking next steps” such as leaving or getting new jobs and relationships or simply using the coping skills they have been taught such as recognizing triggers.

When thinking about how this translates into the logistics of evaluation, the survivors in this group highlighted a tension between experiencing progress as opposed to capturing progress in measures or validated tools that say nothing about their personal journeys, and “it does look different for every woman” (OJG12).

Also identified for some was a particular issue around timeframes with change being something that continued after the groups had ended. “How they felt when it finished and how they feel now a year on, because it's new” (JG07).

Therefore, in thinking about evaluation, survivors signaled the need for room around capturing personal journeys and the tension between that and validated, standardized scales commonly used. It is interesting that none of the women interviewed alluded to the standardized measures routinely collected by the provider and this might speak to their lack of meaning for those whose progress they are meant to capture. An evidence base requires some level of standardization in order to read across studies. Co-produced evaluation work should be able to negotiate a survivor-centered approach that captures their recovery pathway with measures they recognize.

A very practical issue around access to support and healthcare was reported. Survivors in these groups spoke about delay in getting the right help, inappropriate referrals by general practitioners to general therapy groups, and barriers to using ongoing healthcare for fear of examination by male clinicians.

I got a bit stressed out because I was going to this stupid like therapy, it works for everything domestic violence, rape, stress, bipolar, schizophrenia … some tools were good but the rest didn’t apply. (JG06)

if I went for a smear, and it was a man, I couldn’t do it. (JG07)

Therefore, short-term project-related interventions and their evaluation do not match the life course frame that survivors use in relating how they got to the group and what happened subsequently to ongoing support needs. A simple measure of if survivors are getting the right help or the help they need might be a meaningful outcome.

## Discussion

Interviews with survivors of sexual abuse/violence, around their experiences of group support, identified themes around *anger *and its source in *the injustice system* alongside the progress to recovery through shifting the focus of blame away from themselves as victims to the people and systems by which they had been victimized. Part of the contribution of groups to recovery was seen by some as channeling that anger to fuel activism in order to address that injustice. The contribution of using a survivor-led approach has facilitated a theoretical expansion of group recovery pathways, in recognizing the need to channel anger and to envisage an active role for groups to foster collective, socially networked, activism. Other themes contribute to our understanding of how exactly the group setting helps, the group as “*company*” illustrating the potential to create bonding/sharing or solidarity through community, a need documented in earlier research (Konya et al., 2020) and which speaks to the isolation of sexual violence victimization. Unique to this analysis was the framing of the group as a *yardstick* against which survivors plotted their own progress. The study was carried out to inform the evaluation of groups and themes addressing how to capture progress posed the challenge to evaluators around survivors being able to own and recognize their own stories and recovery destinations currently not captured in traditional evaluation outcomes. Standardized measures are generally seen as “what the funders want” and should enable pooling of evidence; however, funding to utilize these data sets is scarce in the charitable sector. Routine outcomes collated by group providers were Likert scales around mental health, self-esteem, anxiety, positive choices, increased skills, increased opportunities, drug/alcohol use, feeling safe, managing changes, isolation, and control but were not mentioned by participants in this study in relation to their own views on evaluating their progress. This may speak to a lack of recognition of current evaluations by those whose progress it is designed to capture. Survivors need to see their own journeys and have more ownership over monitoring/outcome data to ensure greater validity and better use of what is collected.

Unresolved anger and the source of that in retraumatizing criminal justice processes was clear in these survivors’ accounts. Their experiences of being legally silenced are evidenced in UK national figures, with fewer than 1 in 70 rapes reported to the police in 2020, resulting in a charge or summons let alone a conviction (Barr & Topping, 2021). The UK government itself has acknowledged that survivors are being failed (HM Government, 2021). The need to channel this anger into activism, such as helping others or opportunities to represent survivors’ experience, was seen as something groups could facilitate.

The strength of shame and self-blame for survivors of sexual violence/abuse is extensively documented in the literature (Cantor et al., 2015). Victim blaming is linked to widely held rape myths that center on the responsibility of victims to avoid rape and the absence of perpetrator agency (Weiss, 2009). This in turn plays out within the criminal justice systems with a disproportionate focus on survivors’ credibility (Hohl & Stanko, 2015) and helps to explain the large gap between prevalence of sexual violence and convictions or the “justice gap” (Temkin & Krahe, 2008).

The process of shifting blame is already seen as part of recovery (Bonnan-White et al., 2018; Konya et al., 2020). Measures of self-blame have been used in understanding recovery and framed as a mediating factor to ongoing harm such as alcohol use (Peter-Hagene & Ullman, 2018). The visceral recognition of the breakthrough survivors in this study experienced in moving from self-blame toward anger toward the perpetrator or justice system suggests the need for an even greater focus on this process. We have emphasized the need to highlight this element of recovery by using the term *unblaming*, the act of explicitly and repeatedly absolving survivors of agency, which for one survivor meant “I don’t actually feel stupid anymore” (JG07).

Wider societal victim blaming through rape myths is well recognized, so much so that courts require instruction on how to counteract their dominance in rape trials (Crown Prosecution Service, 2021). There is a need to see the experience of sexual violence and its targeting through a wider inequality and intersectional lens (Meyrick, 2022) and not as a series of individual victims experiencing isolated incidents. The burden of victimization and victim blaming is greater for marginalized groups such as racially minoritized women (Sigurvinsdottir et al., 2020). The direct experience of the “injustice system” in this study and the resulting sense of anger show us how social inequalities are played out in individual lives. It would be a mistake therefore to present these data without connecting it directly to patterns of power and inequality (Meyrick, 2022).

The meaning behind the lack of participants from racially minoritized communities in both the specialist sexual violence services and this research tells us that those more likely to be victimized are also marginalized from support services as found in related area-based needs assessments (VOSCUR, 2018). The women in this study perceived multiple layers of silencing, disbelief, and isolation as something groups were able to restore with activism as a way to channel the anger with the inequality at the heart of sexual violence. Transforming pain into power as a pathway to recovery is documented in previous work (Herman, 1998).

Previous research with survivors emphasizes the need to see some acknowledgment of the wrong done to them often not forthcoming when telling family or friends (Ahrens et al., 2010; Bonnan-White et al., 2018) or through legal redress (McGlynn & Westmarland, 2019). The counterweight provided by members of the group listening, believing, and validating was apparent in these accounts and support previous work identifying a changing “kaleidoscope” of definitions survivors use to frame “justice”: consequences, recognition, dignity, voice, prevention, and connectedness (McGlynn & Westmarland, 2019). An interesting and novel understanding from this study is the role of “company” in helping survivors see themselves through, alongside and in comparison to others. There was something important for recovery in groups as a “yardstick” against which survivors were able to recognize their own progress. This included recognizing their own PTSD through hearing others talk about theirs or through being able to see how far you have come listening to others struggling earlier in the process.

Finally, accounts point to the need to situate the experience of survivor groups within a wider life course perspective. Before the group, many struggled to get to the right help, for example, through inappropriate referrals to general mental health support groups. How survivors get to groups or the right help seemed to be an issue around education for health professionals. Others talked about their recovery needs once the group had finished. Survivor's ongoing healthcare needs and the avoidance of triggering healthcare such as cervical screening or intimate examinations require further research. Specialist sexual violence support may therefore need to better educate those likely to refer to them and also reach into health services to advocate for the ongoing needs of their clients.

### Study Strengths and Limitations

This study examines the reports of women attending two “justice groups” provided by SARSAS to support their recovery from sexual violence and abuse. The women interviewed anecdotally experienced the research as very positive, and interviews were carried out within the protective framework of the specialist sexual violence service SARSAS leading to authentic and safe conversations for both participants and researchers. Five of the 18 volunteers did not manage to book interview slots before data collection closed; this lack of participation could signal a negative experience or a need for greater flexibility in carrying out trauma-informed research in a vulnerable group. This study includes certain methodological limitations, as a qualitative study, sample recruitment is not trying to achieve a generalizable subsample of all survivors, and the intention was to include the widest possible range of experiences. All of the group attendees volunteered to be interviewed (*N* = 18), but interviews were completed with only 13 of those due to the inability to book in a time during the data collection period. It might be that those facing the most adversity found it harder to practically engage with the research study. There was a gap between completing the group and taking part in the interviews, which may have altered recall or allowed time for reflection. This could be addressed through a purposive sampling of different timeframes. COVID-19 restrictions forcing group sessions online may have changed the dynamic within them.

Racially minoritized communities, people with disabilities, men, and sexually nonconforming groups are highly targeted by sexual violence but are underrepresented in specialist support service groups and therefore this study. The diversity of the participants in this study was a reflection of attendees of the groups studied and within which the range of minoritized groups was lacking (generally heterosexual [three bisexual and three unknown] White women [one mixed heritage]), a recognized gap in services. The relative absence of voices from marginalized communities refocuses our attention on sexual violence as an intersectional issue that can mean support services lack diverse reach (Imkaan, 2018). In common with other qualitative studies, an informed decision is needed in considering the transferability of our findings beyond the context in which the data were collected. The reported findings relate specifically to the UK context, within a city and mixed city and rural southwest location.

## Conclusions

Group support represents a common model for the provision of support for victims/survivors of sexual violence and abuse. To understand both if and how groups work, we need to be able to evaluate the evidence of their contribution to recovery. This study centered on lived experience as the starting point for recommendations for the evaluation of two groups and interviewed women who had participated. Findings provided in-depth accounts of how groups contribute to reducing a sense of isolation but also help participants’ own understanding of their progress. The level of anger and need to see recovery as something that progresses to activism around wider inequality issues was a powerful message for the researchers. SARSAS have already established an activist/voice group for survivors to channel lived experience into policy and research but on their terms. Reclaiming the agency of consultation work beyond researcher-led and project-based patient and public involvement (PPI) is needed to rebalance power. Specialist sexual violence services should see activism as part of their role and part of an individual’s recovery. SARSAS have evolved and expanded their group offering on the basis of these study findings (https://www.sarsas.org.uk/support-and-information/support-services/group-work/).

A key point of recovery for these participants and therefore for evaluation as a potential measure of progress was shifting blame from the self to the perpetrator and system. It was felt that this process needed emphasis and, through the authors’ previous work with survivor accounts (Higson-Sweeney & Meyrick, 2022), led to the identification of the term “unblaming” as a way of surfacing the need to repeatedly combat ingrained rape myths of responsibility. This could be a measurable outcome of progress and has been used in others.

In contrast, women evidenced a diversity of recovery pathways and spoke to a need for the evaluation of specialist services to be survivor-led and reflect their experiences, against which standardized measures often jarred. A shared vision of evaluation developed through joint understanding of what evaluation is used for seems the best way to address the tension between a common evidence base and outcomes that survivors see themselves in. Patient and public involvement (PPI) in service delivery and evaluation would benefit from real collaboration with those they serve, seeing success through their eyes.

### Key Points

- Anger channeled into activism is a potential focus for ongoing support beyond the groups.
- Framing recovery in the real-world context of inequality and intersectionality is important.
- Lack of diversity in clients of specialist support services is a further barrier to support for marginalized groups.
- “Unblaming” survivors’ needs greater emphasis and evaluation have and could use this as an outcome measure.
- Evaluations need to draw on survivors/service users’ definitions of success.

## Figures and Tables

**Table A1.** Audit Trail Theme to Quotes.

Being angry	OJG4: I could have been going into it thinking, right, I'm going to get a law degree, and I'm going to sue the bastard. OJG04: cause apart from the castration I can't think of much justice, but I don't think there's any courses about castrating people in the UK right now OJG18: I'm starting to get to that stage now. Where I actually feel I do feel angry, I think it's healthy to start feeling angry, I've met spent many years where I just felt just horrible, sad, unhappy, guilty, shame, ashamed, but not angry when I would like to feel angry. So I have occasionally had occasions where I do feel angry. And I think that's good. I don't think something wrong with that. I think that's a healthy way of recognizing that that wasn't right. And you get you need to get to that stage where you feel. I'm not at fault. And it's not right, what happened. But sometimes that can take a very long time. Sorry, I'm waffling on a bit there. I don't know if I've lost the thread of what I was saying. But getting moving myself forward in a way that getting angry helps. I would say yeah, OJG19/1: I guess I'm still in work I've not. I've not smashed the door in the toilet. Nearly made my counselor cry just with, like, exhaust ration of this. Especially Initially, I was having real anger issues. very understandable. All of my anger was really misplaced. So one of the things I was most angry at was automatic door opening buttons. OJG19/2: the ones that are there for someone to press if they're in a wheelchair, and they need the door opened. weapon. Everyone is supposed to open the door. OJG19/3: It's like there I've scanned it in the morning. Scanning out that's all you get. Yeah, I've got a big thing about buttons and doors. But I haven't kicked the door and I haven't smashed it and it's you know it's a whole unit extra year down the line. So that's the success OJG6: like talking about it does help stops you from being so angry, because I never used to really do that. OJG6/2: I'm less angry. Yeah, I can talk about it will be the main thing. OJG9: she just got very angry and started talking about something which made sense, but it just got her it then just started feeding into the anger and getting her more and more wound up to the point where she'd like full on slammed her hands down on the table. And that scared the crap out of me.
Using Anger - activism	OJG08: because we're all angry about it. And even the police are frustrated, you know, just it's not there, obviously, a therapeutic service in a way to kind of offer counseling and group work like this, you know, they're not lobbyists, but they are obviously aware of organizations that are that we can get in touch with if we want to kind of have a bit more focus on that as well. OJG17/1: at the end of the course, I was kind of fed up. I wanted to join SARSAS help them fight and help publicize. You know, and, you know, I did contact them about volunteering. Yeah. And very frustrated. I was very frustrated that they said, Oh, you know, normally we don't take anyone for 12 months after. And I thought, at that stage, I thought, you know, what are they waiting for? I'm fine. And I have to say, I'm having now left it. Six months. I certainly needed it.
	OJG17/ 2: that still is that still is something that I will I would really like to and get involved
	OJG17/3: successful B You know, I was helping on some campaign, you know, doing something or success would be to help other women. Yeah. Yeah. And success would be for me to go global. Attend a big summit. You know, be part of, but anyway, I have things like that in my head. You know, it's i'm not i'm not really being facetious. It's just not necessarily me, but to see it become something that's talked about, and, yeah, you know,
	OJG9: And I'd love to one day, like be able to run one of those groups, if I, like, I'm contemplating a career change. I really want to access assets, and anything that comes up, and they're probably going to get fed up with me. So I'll email them and be like, Can I do this? Like, am I suitable?
Injustice System	OJG02: Then I realized having gone back to a to a group, where we talked about things that were a bit on the side that I'd rather not do again, having not having, in my situation, the outcome for me wasn't very good. Yeah, revisiting the place where I, I've lost my case, essentially. OJG08 ref 1: you know, they told us facts, you know, about what's happening legally today, you know, with getting, you know, people coming forward to tell people to tell the police to get it through the justice system to get it to court to get a conviction, which is like hen's teeth, ref 2: some people, myself included, you know, very angry and passionate about things, and how unfair the justice system is, and how unfair it is that the burden is on us to prove so much. And even the biggest evidence of CCTV sometimes isn't enough and or experts, psychologists are allowed in murder trials to explain behavior and yet experts are not allowed in rape and abuse trials to explain why people react the way they do to trauma, because it will become biased toward the person accused, which is totally unfair. Totally unfair how you can treat a murder trial where someone's life is taken. And a rape trauma or abuse trauma where someone's life is taken, but they're still alive, and you treat them in two different ways. ref 3: justice isn't just about criminal justice, it's about getting your life back. Which that was good that that was a good one that I never thought about, like the real justice is used or being alive and you're getting your life back and you're not letting them win. OJG12 ref 1: finding a way of recovering our lives. Make his part of the difficulties that the women who experience sexual violence is that having the sense of injustice prevents us from car carry on with our lives. OJG14: at the end of the day, a lot of this, these problems don't get justice. OJG16: So my understanding was, that it was, the aim was to help me find my own sense of justice. So even though I've not had justice through, you know, the normal systems, with what people would think of as Justice, it was more to feel that within myself, I could find that kind of peace within myself, that I've got justice for myself. So I guess that's the sad bit that I didn't feel I got to that point because of the great time to end earlier than expected. OJG18: I don't think any of the women there have got justice, I can't think of any of them that got justice. But looking for our owns a sense of justice.
	OJG18/2: you aren't going to get closure, as they say, or very likely, the perpetrator isn't gonna turn around and say, I'm sorry, I did it, I'll go to prison and whatever, they're just not gonna OJG18/3-4: The whole process of I mean, not everyone had gone through the criminal justice system. But that whole process, if they had is horrific, and then fulfilling to women who hadn't gone through that and feeling maybe they should have, it's just like, everything's like, really difficult to process. So I think that they were trying, I think the facilitators were trying to help people feel as though they could get a sense of justice without that thing where the perpetrator would accept responsibility or go to prison or whatever. Because that's not going to happen 99% of the time.
	OJG18/5: my sense of justice would be, if the perpetrator, I mean, there's actually more than one perpetrator, if they weren't going to prison, which they aren't men, CPS was gonna charge them, but the, if my husband would believe me, and support me, that sense of justice, but it's not going to happen, or I need to get to the stage where I know it's not gonna happen.
	OJG19/1: But but they were basically saying, Yeah, unless, you know, unless it's caught on camera or someone else into the prosecute rather than it's not illegal.
	OJG19/2: I think it's just the Justice word. Because I mean, so many people, there is no justice anyway. And certainly, for me, there isn't in any way on that legal side. I mean, to some people, it's because they try and fail some trial, etc. But it's just the connotations of the word
	OJG6/1: I already reported it and nothing was done. poxy caution or bound over for a year was all that was done.
	OJF6/2: destroyed after this many years. And it's like, well, shouldn't be destroyed. But she said, I can write…… And she goes, I don't understand why it's got a caution on there. I go, Well, I don't know. You tell me you’re the police. I can't answer that. He says, well, it wouldn't be nowadays well, nowadays doesn't really help me Does it? I said, I don't have any closure on that either. Because you screw me over or that you screwed me over about the closure.
	OJG6/3: So the money doesn't bother me is the fact that more than anything else? I want an admission that they screwed me.
	yeah, they gave me that closure and that justice that I needed. So we've gone through from when I reported it at 17. I've gone 30 years, nearly my next year, it'll be two years since I reported out. Not
	OJG6/4: Because no one record that. That that was what was done. This is what was done. Right. You had an injustice done by him and then we added by giving you another injustice and not giving you that closure and that acknowledgement that this had happened.
	OJG6/5: I guarantee he's done it again, more than one, I can guarantee because he got a lot more cocky as well, because he'd been reported, he admitted. And they just said, well done you. We're gonna give you a slap on the wrist Now, don't get in any more trouble or getting caught again. So he's going to be more careful.
	OJG7/1: if you go by Justice, then we failed. Because I think out of the group, only one actually got to court and got her conviction. Yeah. And that took years.
Getting angry shifting blame	OJG2/1-2: not to feel a victim not to feel that it was your fault. That was the hardest thing to get, from my point of view, to get in myself. I think my thought process was, in the beginning was all my fault, I should have done, you know, you know, blah, blah, blah, I blame myself completely. But over the years, and it's been certainly since I divulged my situation, to the police. And of course, going forward, you do actually start to believe that it actually wasn't your fault. And you do gain a lot of confidence from that. And it being said to you over and over and over again. And then you've got the research that proves that there's a profile on these, these people that are, you know, these horrible people who are out to target you and they recognize your failures or your insecurities. They prey on that you might, from that point of view, it, it does help make you more confident and make you feel that you're not. It's not the biggest thing over the years OJG2/3: think that's, you know, that is the goal is to hit the main goal is is not your fault. And it's definitely not your fault. There are people out there who have real bad people. And you've just been unfortunate in your lifetime and your family, you know, groups that you've managed to slip through the net, I suppose, not being recognized, and, you know, people, horrible people will prey on that goal is to recognize that you're not, you know, that you are not that you're not a bad person, and you're not in life as a baby to become a victim of abuse, like you have been. So it's that sort of network of support.
	OJG12/1: the problem is of a different nature is not something that I did wrong.
	OJG12/2: I was able to put the responsibility where it belongs. You know, we the perpetrators, the institutions that support them instead of supporting us, you know, yeah.
	OJG16/1: I've always been stuck with this feeling that it was all my fault that there's something wrong with me. Not really understanding until I went to the group that you know, these were trauma, trauma responses.
	OJG16/2: Going away feeling more compassionate toward myself? Yeah, feeling like it's not my fault. And feeling heard and less isolated?
	OJG6/1: it's about the control the power they do. They don't have power, and they don't have compulsion over that goal, because they can make that and so that's going to bore them. They need they need the sexual attraction bit. They need the compulsion. They need the control and they need that they need all of that to get off.
	OJG7/1: because I felt after what happened to me, I felt so stupid. I should have protected myself more, I should have been aware of what was happening to me.
	OJG7/2: the role of the person that did it to me, not me. Yeah. Yeah. So it helped me with that helped me realize that how we realized that I wasn't going out around in public with like victim written on my forehead.
	OJG09/1: And also, that it really wasn't my fault. I wasn't to blame any less of a person for it happening to me. So that was, that just made me feel so much more comfortable in myself.
	OJG09/2: I don't feel so ashamed of myself anymore. That's such a huge thing. I don't feel like I'm dirty or I'm horrible, or loads of other horrible words that I guess, label myself as for having gone through that I don't feel like that anymore.
